# IL‐33 Elicits LTC_4_ Synthesis in Allergic Inflammation via ST2‐Mediated Activation of Eosinophils

**DOI:** 10.1002/eji.70156

**Published:** 2026-02-28

**Authors:** Vitória F. Rosário‐Garcia, Ericka Guimaraes‐Ferreira, Yasmin Brito‐Leite, Jamille F. Oliveira, Julia Santos‐da‐Silva, Natália R. T. Amorim, Valdirene S. Muniz, Lukas Bolini, Miriam B. F. Werneck, Claudio Canetti, Bruno L. Diaz, Christianne Bandeira‐Melo

**Affiliations:** ^1^ Laboratório de Inflamação Instituto de Biofísica Carlos Chagas Filho Universidade Federal do Rio de Janeiro Rio de Janeiro Brazil; ^2^ Laboratório de Imunofarmacologia e Inflamação Instituto de Ciências Biomédicas Universidade Federal do Rio de Janeiro Rio de Janeiro Brazil

**Keywords:** eosinophil, IL‐33, leukotriene C_4_, lipid droplet, prostaglandin D_2_, ST2 receptor

## Abstract

Like pieces of a puzzle, IL‐33, eosinophils, and cysteinyl leukotrienes seem to come together and orchestrate allergic inflammation. While the IL‐33/ST2 receptor axis is known to activate some classical eosinophil functions, its ability to specifically trigger LTC_4_ synthesis remains elusive. Here, employing a murine model of allergic inflammation, ST2 activation emerged as a key step to LTC_4_ synthesis, primarily achieved by lipid bodies‐enriched eosinophils. Concurring, exogenous IL‐33 elicited LTC_4_ synthesis from activated eosinophils, both in vivo and from human cells in vitro. Thus, relevant to eosinophil‐regulated environments, such IL‐33/ST2‐driven cellular effect may bear therapeutic potential as a target in cysteinyl leukotrienes‐mediated conditions.

## Graphical abstract

Allergic reaction‐derived IL‐33 activates ST2 to trigger selective eosinophil activation. IL‐33 in a type 2 immune response environment induces biogenesis of lipid bodies in eosinophils, platforms for the generation of lipid mediators, and selectively triggers LTC_4_ production.

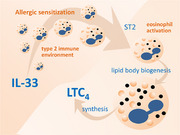

## 1 Introduction

Groundbreaking findings have broadened the comprehension of eosinophil functions from inflammation‐related deleterious roles to homeostatic and tissue repair activities [1, 2]. Such “bad” *versus* “good” functional plasticity is strongly linked to the strategic tissular location of eosinophils [1, 2]; therefore, the characterization of the capacity of locally‐derived molecular factors to regulate eosinophil activity is of particular interest. IL‐33 has been identified as an alarmin‐type cytokine expressed mainly by structural components of various tissues, such as epithelial, endothelial, and fibroblast‐like cells [3]. IL‐33 displays numerous immune regulatory functions, remarkably, the induction of allergic immunological responses characterized by robust eosinophilic inflammation [4, 5], which is recurrently associated with cysteinyl leukotrienes (cysLTs; LTC_4_/D_4_/E_4_)‐driven symptomatology [6]. Of note, most allergic reactions‐produced cysLTs are eosinophil‐derived [6, 7]. Indeed, one of the most distinctive eosinophil effector functions is the lipid body‐compartmentalized enzymatic machinery for inducible LTC_4_ synthesis [8]. However, the mechanism by which IL‐33 triggers such pivotal eosinophilic activity has been overlooked. Our hypothesis anticipates that IL‐33 is an allergic inflammation‐related signal responsible for activating LTC_4_ synthesis within eosinophils, since (1) both tissue resident and infiltrating eosinophils may be directly exposed to tissue‐derived IL‐33 under allergic inflammation; (2) functional IL‐33 receptors ST2 are present on eosinophil surface [9, 10]; (3) eosinophils are “professional” LTC_4_ synthesizing cells [8]; and (4) activation of IL‐33/ST2 axis is known to trigger a variety of other functional activities in eosinophils. Of common knowledge, stimulation of ST2‐expressing eosinophils by IL‐33 promotes survival, degranulation, superoxide anion secretion, and cytokine production [9, 10, 11, 12]. So here, to assess a potential key component of the mechanisms involved in the therapeutic success of targeting the eosinophilic IL‐33/ST2 axis in the allergic inflammatory context [5, 13, 14], we evaluated whether IL‐33 is capable of inducing LTC_4_ synthesis by eosinophils.

## 2 Materials and Methods

### 2.1 Induction of Allergic and IL‐33‐induced Eosinophilic Inflammation in Mice

For either allergic reaction‐ or IL‐33‐induced eosinophilic inflammation, BALB/c or eosinophil‐deficient on BALB/c genetic background mice (∆dblGATA mice; obtained from Dr. Flavio Almeida Amaral at the Department of Biochemistry and Immunology of the Federal University of Minas Gerais, Brazil) [15] were sensitized, as previously described, with a subcutaneous (sc) injection (200 µL) of ovalbumin (OVA; 50 µg; Sigma‐Aldrich) and Al(OH)_3_ (5 mg) in 0.9% NaCl solution at Days 1 and 7 [16, 17, 18, 19, 20]. On Day 14, both *naïve* (non‐sensitized) and previously sensitized mice (as indicated) received an intrapleural (ipl) injection of 100 µL of either allergen (OVA; 50 µg/cavity), IL‐33 (0.1 µg /cavity; Peprotech), or their vehicle (sterile saline). To specifically address the involvement of activation of IL‐33/ST2 pathway in allergic inflammation‐induced eosinophil‐driven LTC_4_ synthesis, sensitized mice were treated with ipl injections of inhibitory molecule sST2 (soluble ST2 receptor at 2 µg/cavity; R&D Systems) immediately before allergic challenge. 4 or 24 h after ipl challenges, pleural cavities were rinsed with 0.5 mL of HBSS (Hank's balanced salt solution). The analysis of pleural fluid cellularity by light microscopy using Neubauer chambers and Panoptic kit‐stained cytospin slides was performed in a blinded fashion by at least two researchers. Pleural cell‐free supernatants were stored at ‐80°C. All animal care and experimental protocols used in this study were approved by the “Animal Use Ethics Committee” of the Federal University of Rio de Janeiro, Brazil (CEUA036‐23/CCS/UFRJ).

### 2.2 *In Vitro* Stimulation of human Eosinophils by IL‐33

Human eosinophils (3 × 10^6^ cells/mL) were isolated by immunomagnetic negative selection from healthy donors blood as previously described [21], after all procedures were approved by the Ethics Review Board (CEP IPPMG/UFRJ 6.324.927) and informed consent was obtained from all participants. Eosinophils were incubated in Ca^2+^/Mg^2+^ HBSS (HBSS^+/+^; pH 7.4) with rhIL‐33 (1 or 100 ng/mL; Peprotech), rhCCL11 (eotaxin‐1 at 5 or 100 ng/mL; Peprotech) or a combination of suboptimal concentrations of rhIL‐33 (1 ng/mL) plus CCL11 (5 ng/mL) for 1 h (37°C). Supernatants were collected and cells were stained as described below. Experiments were repeated at least three times with eosinophils purified from different donors.

### 2.3 Analysis of Lipid Bodies, Lipid Mediator Synthesis, and Cytokine Production

As previously described [17, 18, 19, 20, 21], to quantify lipid bodies, cells were cytocentrifuged (450 rpm, 5 min) onto glass slides. While still moist, cells were fixed in 3.7% formaldehyde (diluted in Ca^2+^/Mg^2+^–free HBSS [pH 7.4]), rinsed in 0.1 M cacodylate buffer (pH 7.4), stained with 1.5% OsO_4_ for 30 min, rinsed in distilled H_2_O, immersed in 1.0% thiocarbohydrazide for 5 min, rinsed in 0.1 M cacodylate buffer, restained with 1.5% OsO_4_ for 3 min, rinsed in distilled water, and then dried and mounted. Cell morphology was observed, and lipid bodies were enumerated in all orthogonal optical sections under light microscopy. A total of 50 consecutive cells (either eosinophils or mononuclear cells/slide) were subjected to blinded evaluation of their content of cytoplasmic osmiophilic lipid bodies by more than one individual. Results were expressed as the mean number of lipid bodies per cell for each animal/condition.

Cytokines (IL‐33 and IL‐5) and the lipid mediators PGD_2_ and cysteinyl leukotrienes LTC_4_/D_4_/E_4_ (cysLTs; as markers of intracellular LTC_4_ synthesis) found either in cell‐free pleural fluids or human eosinophil supernatants were detected by commercial ELISA or EIA kits, according to the manufacturer´s instructions (R&D Systems and Cayman Chemical Co., respectively).

### 2.4 Data Analysis

Results show mean ± SEM for each group. Experiments were performed independently at least twice with 4–6 mice per group, as depicted in the scatter plot graphs (excepting the IL‐33‐injected assay in ∆dblGATA mice, which was performed once). Scientists were blinded to the identities of the groups for the analysis of all parameters (cellularity, lipid body biogenesis, and mediator production); however, they were not blinded during the treatment of the animals.

All parameters analyzed followed a normal distribution and are homoscedastic; therefore, *in vivo* data were analyzed by one‐way ANOVA followed by the Student–Newman–Keuls test. *In vitro* data were analyzed by paired *t*‐test for experimental designs with only two groups by one‐way ANOVA followed by the Student–Newman–Keuls test for more than two groups. Differences were considered significant when *p*  <  0.05.

## 3 Results and Discussion

Understanding the full complexity of eosinophil activation within allergic inflamed tissues is fundamental to opening new avenues for allergy therapeutics. Activation of infiltrating eosinophils by *in situ* secreted molecules is a critical feature in the pathogenesis of allergic diseases. On this note, the role of tissue‐derived alarmins in regulating key allergy‐relevant eosinophil functions, such as cysLTs production, demands further characterization. We hypothesized that IL‐33 secreted at mucosal sites of allergic inflammation would activate eosinophils to synthesize and release the allergy‐relevant mediator LTC_4_. Therefore, concurring with the knowledge that IL‐33/ST2 axis is known to activate eosinophils, this study investigated whether IL‐33 is capable of directly evoking lipid‐body‐driven LTC_4_‐synthesizing machinery within eosinophils and if the activation of ST2 receptors participates in the induction of LTC_4_ synthesis by eosinophils at the inflammatory site of allergic response.

**FIGURE 1:**
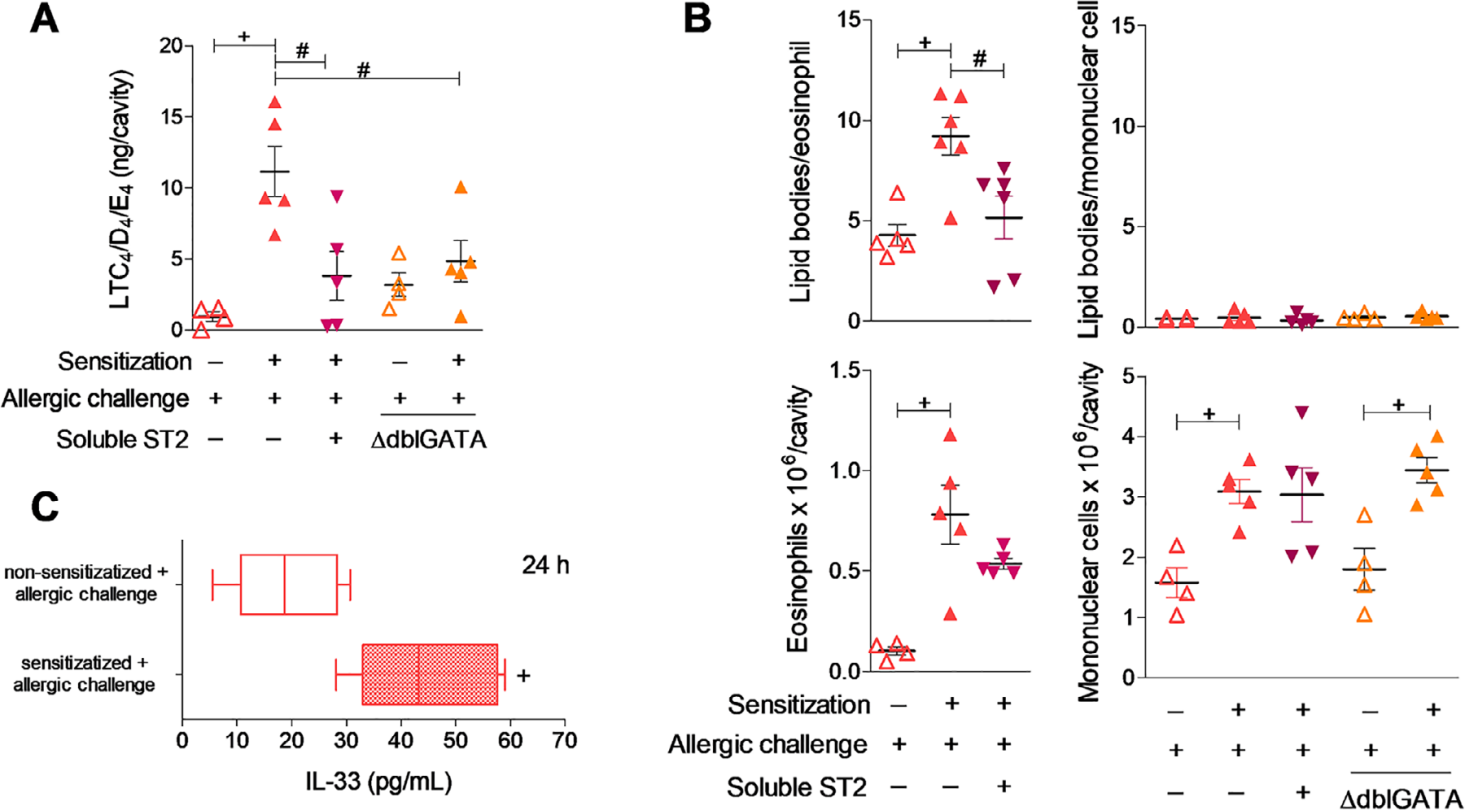
ST2 activation mediates allergic inflammation‐elicited eosinophil lipid body biogenesis and eosinophil‐driven LTC_4_ synthesis. Non‐ and sensitized BALB/c or ∆dblGATA mice (as indicated) were challenged with an i.pl. injection of ovalbumin (12 µg*/*cavity). Treatment with soluble ST2 was performed *in situ* (2 µg/cavity) and all analyses were done 24 h after allergic challenge. A shows cysLTs levels (LTC_4_/D_4_/E_4_) found in pleural cavities after allergic challenge. B displays pleural numbers of eosinophils and mononuclear cells (namely a mixed population of macrophages, lymphocytes and mast cells) and the lipid body content of these cells. C shows the pleural amounts of IL‐33. In A and B, individual animal values and mean ± SEM are shown (*n* = 4–6 per group, as indicated). In C, the interquartile ranges and medians are shown (*n* = 4). Data are from one representative experiment (independent experiments showing the same phenomenon are shown in Figure S2A). ^+^ *p* ≤ 0.05 compared with ovalbumin‐challenged non‐sensitized mice and #*p* ≤ 0.05 compared with ovalbumin‐challenged sensitized mice.

As previously described [18, 19], and shown in Figure 1A, ipl allergic challenge of pre‐sensitized mice induces a significant increase in cysLTs levels associated with a marked pleural eosinophil accumulation (Figure 1B), compared to non‐sensitized mice. Moreover, such infiltrating eosinophils are activated as indicated by their increased cytoplasmic content of lipid body organelles (Figure 1B).

Eosinophil lipid bodies are highly active intracellular sites of lipid mediators synthesis [8] with important roles, not only in tissue homeostasis, but also in allergic inflammation. Their biogenesis is a hallmark of eosinophil activation and preparedness to engage the mobilization of polyunsaturated fatty acid precursors and the enzymatic cascade that leads to the generation of a variety of lipid mediators, which play central roles in allergic symptoms. Specifically, eosinophil lipid bodies are known to be the intracellular compartments of LTC_4_ synthesis in these allergic conditions, as established by directly detecting eosinophils actively producing LTC_4_ through lipid immunolocalization assays (EicosaCell) [19, 22]. Eosinophil lipid body‐driven LTC_4_ synthesis and the subsequent extracellular generation of LTD_4_ and E_4_ activate airway immune and structural cells, driving type 2 immune responses and mucus secretion in the lungs, thus exacerbating respiratory allergic diseases, such as asthma [6].

Here, ST2/IL‐33 axes' role as a mediator of allergic inflammatory response was expanded to also contribute to loading eosinophil cytoplasm with newly assembled lipid body organelles, previously immune‐detected as the intracellular sites of eosinophil LTC_4_ synthesis in allergic conditions [18, 19]. As shown in Figure 1A, such lipid body‐enriched eosinophils were further confirmed as the LTC_4_‐synthesizing cells, since allergic reaction‐induced LTC_4_ synthesis is abolished in ∆dblGATA animals, which are deficient in eosinophils (Figure 1A). In alignment with our hypothesis, these activated LTC_4_‐synthesizing eosinophils are immersed in an IL‐33‐enriched inflammatory environment (Figure 1C), which could therefore represent the responsible stimulus for a direct activation of the LTC_4_‐synthesizing machinery in the pleural infiltrating eosinophils. Hence, soluble ST2—an inhibitory molecule that competes with the cell surface‐expressed ST2 receptor—was administered locally (ipl) immediately before the allergic challenge. As shown in Figure 1, the ST2 decoy receptor employed here did inhibit eosinophil activation as revealed by diminished allergic inflammation‐elicited LTC_4_ synthesis (Figure 1A) as well as eosinophilic lipid body counts (Figure 1B), despite not affecting eosinophilia magnitude at the allergic site (Figure 1B). Neither allergic inflammation nor the ST2 treatment altered the negligible basal content of lipid body organelles within resident mononuclear cells found in pleural cavity (Figure 1B). And strengthening the dissociation between ST2‐driven LTC_4_ synthesis and these other non‐eosinophilic potential cell sources of LTC_4_ during allergic inflammation, such pleural population of mononuclear cells grew slightly (but significantly) in size, independently of either IL‐33‐driven ST2 activation or eosinophil modulation (Figure 1B).

**FIGURE 2:**
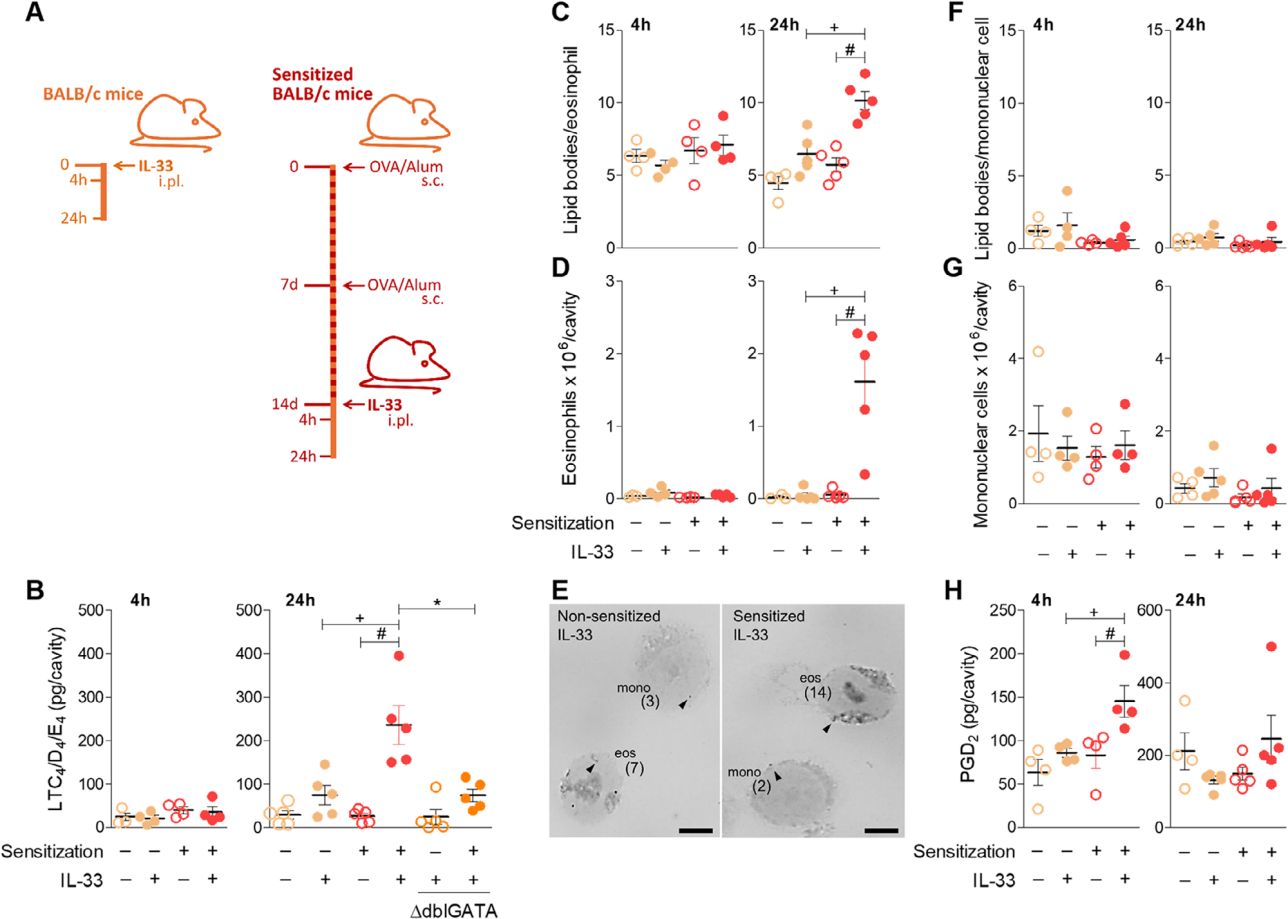
IL‐33 induces LTC_4_ synthesis associated with eosinophil accumulation and lipid body biogenesis in sensitized BALB/c mice. A shows brief schematic representations of pleurisy models induced by ipl injection of IL‐33 in non‐sensitized and sensitized mice, as indicated. Mice received ipl injection with IL‐33 (0.1 µg/cavity) and pleural fluids were analyzed within 4 or 24 h, as specified in each graph. B shows cysLTs (LTC_4_/D_4_/E_4_) levels found in cell‐free pleural fluids. C and D show number of cytoplasmic lipid bodies within eosinophils and pleural eosinophil counts, respectively. In E, representative images of osmium‐stained pleural cells found 24 h after IL‐33 stimulation are shown (arrow heads indicate single lipid bodies; numbers in parentheses depict total lipid body counts performed under light microscopy in orthogonal optical sections of each cell; eos = eosinophil; mono = mononuclear cell; bar = 5 µm). In F and G, number of cytoplasmic lipid bodies within mononuclear cells and pleural mononuclear cell counts are shown. H displays PGD_2_ levels found in cell‐free pleural fluids. Individual animal values and mean ± SEM are shown (n = 4–5 per group, as indicated). Data are from one representative experiment of 3 separately performed experiments (excepting data related to IL‐33‐injected ∆dblGATA mice, which was done once). An independent experiment showing the same phenomenon is shown in Figure S2B. ^+^ *p* < 0.05 compared to IL‐33‐challenged non‐sensitized mice; #*p* ≤ 0.05 compared with saline‐challenged sensitized mice; *p ≤ 0.05 compared with IL‐33‐challenged sensitized BALB/c mice.

The inability of local treatment with soluble ST2 to significantly modify eosinophil accumulation in this particular experimental model of allergic inflammation does not preclude a role for IL‐33 in the allergic response as a whole, including events that either precede the antigen challenge or take place in other sites as result of the subcutaneous sensitization [23, 24]. Nonetheless, especially related to our study is that IL‐33 is a pleiotropic tissue‐derived signal with well‐established eosinophil‐activating effects. So, to investigate whether exogenous IL‐33 can promote the activation of recruited eosinophils and, therefore, control eosinophil LTC_4_‐synthesizing machinery in vivo, we developed a mouse model of IL‐33‐induced eosinophilic inflammation in previously sensitized mice (Figure 2A). We initially observed that local administration of IL‐33 (0.1 µg/pleural cavity) to *naïve* non‐sensitized mice did not elicit an allergy‐like inflammatory response, failing to trigger LTC_4_ synthesis (Figure 2B) or eosinophil migration/activation (Figure 2C,D), either 4 or 24 h after IL‐33 injection. In contrast, ipl challenge with IL‐33 into sensitized mice did induce pleural eosinophil accumulation within 24 h (Figure 2D), but not as early as 4 h (Figure 2D). Recruited eosinophils showed elevated numbers of cytoplasmic lipid bodies (Figure 2C,E) simultaneously with increased pleural levels of cysLTs LTC_4_/D_4_/E_4_ within 24 h (Figure 2B). The enhanced IL‐33‐driven LTC_4_ synthesis *in vivo* seemed to originate from lipid body‐enriched activated eosinophils, since IL‐33 failed to elicit *in vivo* synthesis of LTC_4_ within sensitized ∆dblGATA eosinophil‐deficient mice (Figure 2B). Moreover, the remaining leukocytes at the inflammatory site, the pleural population of mononuclear cells (Figure 2G), did not show signs of activation. As shown in Figure 2E,F, the pleural mononuclear cells did not show increases in their cytoplasmic content of lipid bodies in response to IL‐33 stimulation.

Distinct from previous observations regarding other eosinophilic conditions, such as pleural allergic reaction [17, 20], IL‐33‐induced lipid mediator synthesis appears somewhat selective towards LTC_4_. As shown in Figure 2E, IL‐33 failed to promote PGD_2_ production within 24 h, while establishing an LTC_4_‐synthesizing eosinophilic reaction. Strikingly, IL‐33 was capable of inducing PGD_2_ synthesis at 4 h in sensitized mice. Since there is no pleural eosinophilia in this earlier time point (Figure 2B), the cellular source of the increased pleural PGD_2_ amounts detected within 4 h of IL‐33‐induced pleurisy in sensitized mice (Figure 2D) clearly does not correspond to eosinophils. While the IL‐33‐stimulated cell type responsible for this acute PGD_2_ production was not characterized, PGD_2_ is a known product of resident pleural mast cells activation [17].

In our IL‐33‐elicited mouse model of eosinophilic inflammation, IL‐33‐driven direct effects only became apparent in previously sensitized mice that responded to the alarmin stimulation *in vivo* with eosinophil migration, lipid body biogenesis, and LTC_4_ synthesis at the injection site. *Naïve* mice did not respond to IL‐33 with eosinophil recruitment, thus precluding any study on eosinophil activation under this condition. While IL‐33 did not seem to play a relevant role in the accumulation of eosinophils triggered by antigen (Figure 1B), probably due to the poly‐mediated nature of allergic inflammation, it emerged as an essential signal in the activation of LTC_4_ synthesis by recruited eosinophils. Hence, the role played by this alarmin requires a compliant environment skewed toward type 2 immunological responses. The characterization of the specific changes brought by sensitization in promoting an allergic profile should be the subject of further investigation. These changes may involve differential expression of ST2 among resident cells with an indirect role for IL‐33 in eosinophil mobilization and activation through ST2 engagement. ST2 is widely expressed among allergy‐related immune cells upregulated upon allergic challenge and may contribute with additional signals that act in a concerted fashion to activate eosinophil production of LTC_4_. Whether sensitization by itself specifically changes ST2 pleural levels is still elusive, the mechanism involved in sensitization‐dependent IL‐33 ability to trigger LTC_4_ synthesis by eosinophils appears to be broader. Indeed, this same sensitization strategy was also required by exogenous PGD_2_, CCL11, or leptin to trigger eosinophilic inflammation [17, 18, 19]. As shown in Figure S1, the sensitization per se assembles a systemic eosinophilic environment (within 14 days; time point of IL‐33 administration), with enhanced bone marrow production of eosinophils and blood eosinophilia associated with increased serum levels of IL‐5, but with no alterations of IL‐33; an environment which would allow stimuli like IL‐33 to evoke an eosinophilic reaction.

**FIGURE 3:**
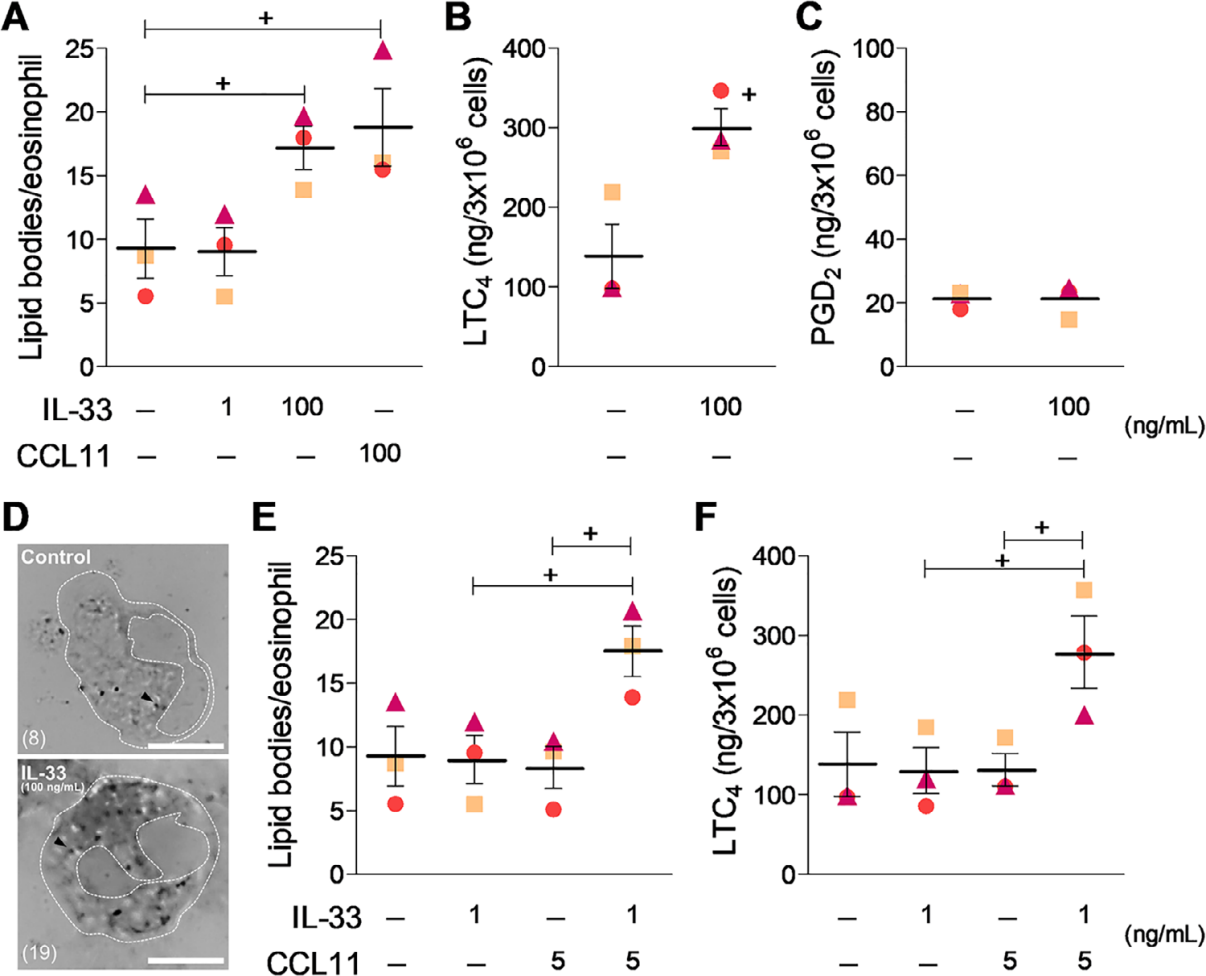
IL‐33 directly activates LTC_4_ synthesis by human eosinophils *in vitro*. Freshly peripheral blood‐isolated human eosinophils were stimulated with IL‐33 (1 or 100 ng/mL) or CCL11 (5 or 100 ng/mL), as indicated. In vitro analyses of lipid body biogenesis (A, D and E) as well as the generation of LTC_4_ (B and F) and PGD_2_ (C) in cell‐free supernatants were performed 1 h after stimulation. In D, representative images of osmium‐stained human eosinophils are shown (arrow heads indicate single lipid bodies; numbers in parentheses depict total lipid body counts performed under light microscopy in orthogonal optical sections of each cell; dotted lines highlight nuclear and cellular limits; bar = 5 µm). Results are expressed as the means ± SEM from three distinct experiments with eosinophils purified from different donors, each one depicted by a different colored symbol (squares, triangles, and circles). ^+^ *p* < 0.05 compared with control group.

In addition to its *in vivo* effect (Figures 1 and 2), here we discovered that IL‐33 is also able to prompt *in vitro* LTC_4_ synthesis by human eosinophils, unveiling IL‐33's ability to directly trigger the assembly of an effective lipid body‐driven LTC_4_‐synthesizing machinery in eosinophils (Figure 3). The *in vitro* assays showed that the IL‐33‐induced direct activation of eosinophils was an acute phenomenon (detected within 1 h) dependent on concentration (Figure 3). At 100 ng/mL, IL‐33 achieved the same level of eosinophil activation as that induced by CCL11, a rapid inducer of biogenesis of newly formed cytoplasmic lipid bodies within eosinophils (Figure 3A,D). Subthreshold concentrations of these two stimuli used in combination triggered the biogenesis of new organelles within IL‐33/CCL11‐stimulated eosinophils (Figure 3E). And like the *in vivo* findings, *in vitro* stimulation by IL‐33 evoked release of de novo synthesized LTC_4_, but not PGD_2,_ from activated eosinophils (Figure 3B,C); an acute phenomenon (1 h), that occurs simultaneously to the observed increase in the cytoplasmic numbers of LTC_4_‐synthesizing compartments within activated eosinophils. Eosinophil LTC_4_ synthesis was triggered by either IL‐33 itself (100 ng/mL; Figure 3B) or by the synergism of suboptimal concentrations of IL‐33 and CCL11 (Figure 3F). Of note, previous unsuccessful attempts to demonstrate the stimulatory ability of IL‐33 to trigger LTC_4_ synthesis by eosinophils, although using similar concentrations of the alarmin, measured LTC_4_ 18 h after IL‐33 stimulation [10]. LTC_4_ is generated rapidly by eosinophils, reaching maximal levels between 15 and 30 min after exposure to stimuli, and is highly unstable in biological fluids with no evidence of delayed generation [25, 26, 27].

Altogether, IL‐33‐induced eosinophil activation culminates with enhanced synthesis of cysLTs, unveiling a new IL‐33‐driven mechanism of proallergic effect, which may correspond to a previously unappreciated key component of the beneficial impact of IL‐33‐targeted therapy [28]. Our findings reinforce IL‐33 as an attractive target for antiallergic treatments in the management of allergic inflammatory diseases, importantly, in patients with refractory responses to conventional approaches.

## Conflicts of Interest

The authors have no conflicts interests.

## Supporting information




**Supporting File**: eji70156‐sup‐0001‐figureS1‐2.pdf.

## Data Availability

All data points are shown individually in the figures together with mean and SEM.
