# Supplementary material for: IL‐33 Elicits LTC4 Synthesis in Allergic Inflammation via ST2‐Mediated Activation of Eosinophils
Source: Eur J Immunol. 2026 Feb 28;56(3):e70156. doi: 10.1002/eji.70156 (PMC12949486; doi:10.1002/eji.70156)
Supplement: Supplementary file 1 — Supporting File: eji70156‐sup‐0001‐figureS1‐2.pdf. [file EJI-56-e70156-s001.pdf]

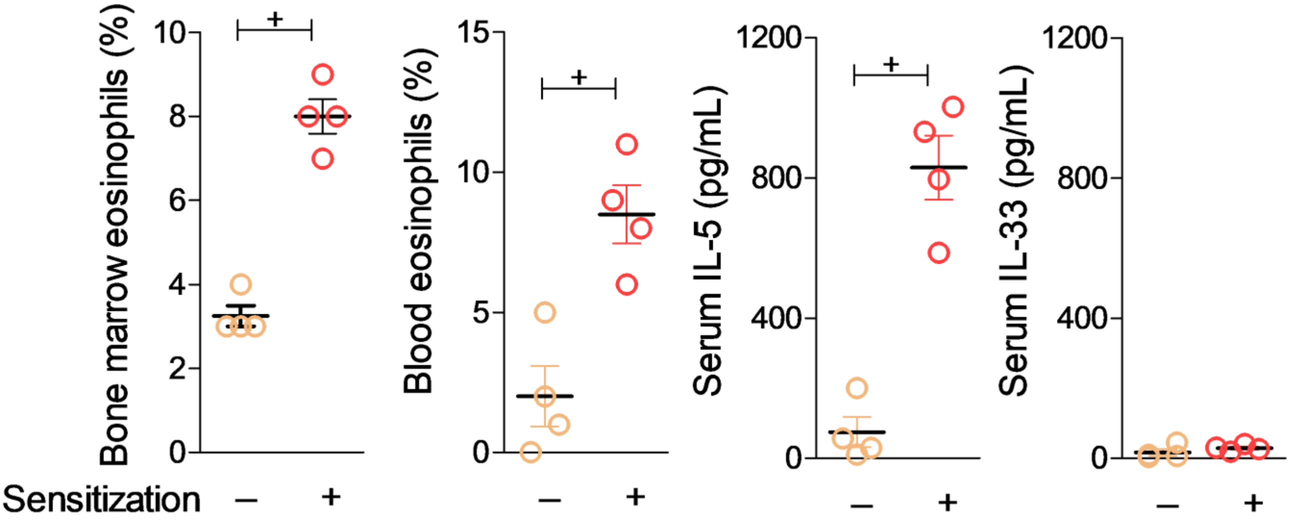

Supplementary Figure 1. Sensitization *per se* triggers a systemic eosinophil-skewed environment. Numbers of bone marrow eosinophils, circulating eosinophils, as well as IL-5 and IL-33 serum levels are shown from non- *versus* sensitized BALB/c mice. Analysis was performed within 14 days of subcutaneous sensitization. Individual animal values and mean ± SEM are shown (n = 4 *per* group, as shown). The experiment was performed once. $^{+}p < 0.05$ compared to non-sensitized control animals.

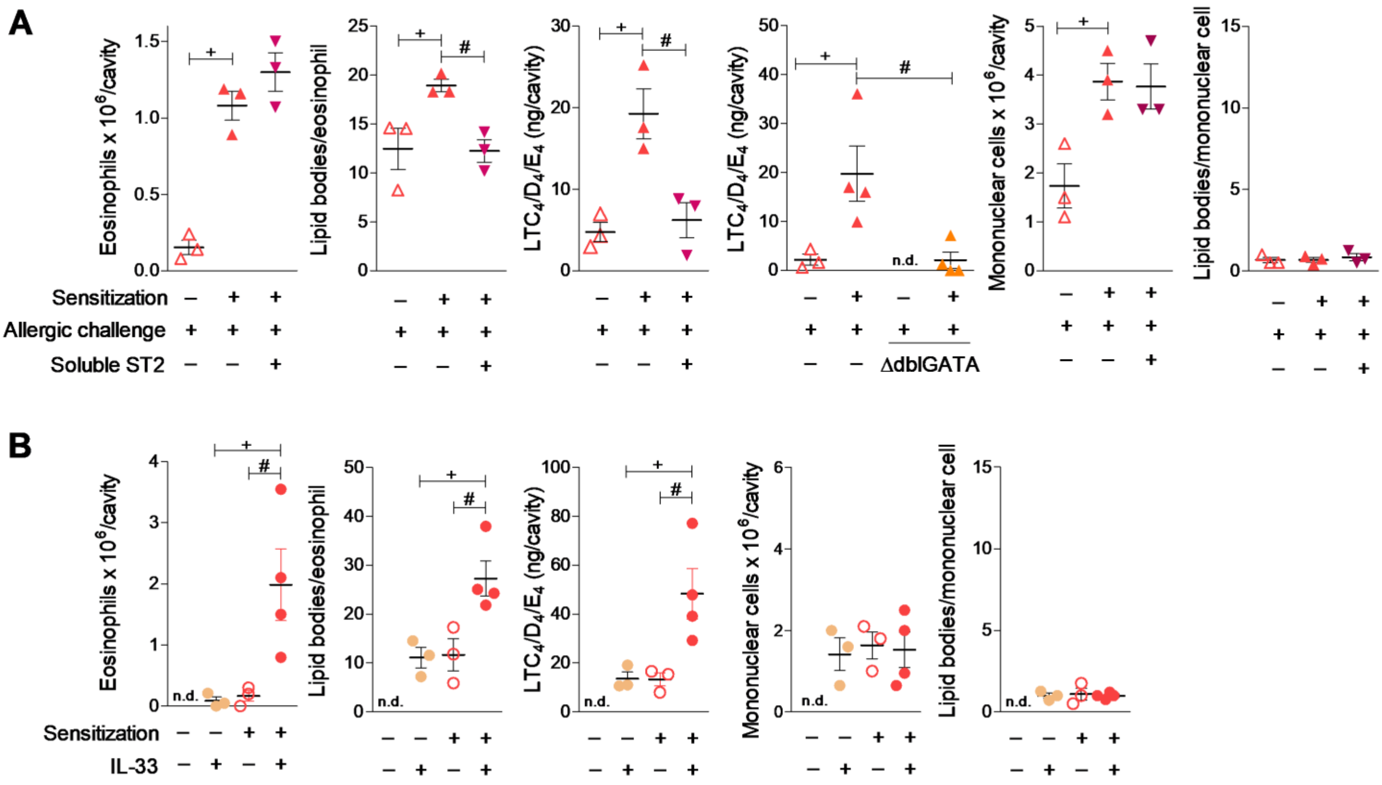

Supplementary Figure 2. Independent experiments reproduce the same key phenomena presented in Figures 1 and 2. **A** shows the effect of IL-33 signalling inhibition with sST2 and eosinophil deficiency during an allergic response-induced eosinophil activation and LTC_4 synthesis (also shown in **Figure 1**). In **B,** selective eosinophil activation and LTC_4 synthesis are triggered by IL-33 challenge in previously sensitized mice (also shown in **Figure 2**). Analyses were performed 24 h after intrapleural challenge with allergen (**A**) or IL-33 (**B**). Individual animal values and mean ± SEM (n = 3-4 *per* group, as indicated) are shown. + and # show $p \leq 0.05$ for the indicated comparisons.
